# Sample size calculations in clinical research should also be based on ethical principles

**DOI:** 10.1186/s13063-016-1277-5

**Published:** 2016-03-18

**Authors:** Bruno Mario Cesana, Paolo Antonelli

**Affiliations:** Unit of Biostatistics and Biomathematics, University of Brescia, Viale Europa 11, 25123 Brescia, Italy

**Keywords:** Sample size calculation, Statistical power, Confidence limits power, Delimited confidence intervals, Ethical principles

## Abstract

Sample size calculations based on too narrow a width, or with lower and upper confidence limits bounded by fixed cut-off points, not only increase power-based sample sizes to ethically unacceptable levels (thus making research practically unfeasible) but also greatly increase the costs and burdens of clinical trials. We propose an alternative method of combining the power of a statistical test and the probability of obtaining adequate precision (the power of the confidence interval) with an acceptable increase in power-based sample sizes.

## Correspondence

Jia and Lynn [1] describe a sample size calculation based on an “approach that considers both statistical significance and clinical significance.” The power-based sample size for a statistical test is iteratively increased until there is a satisfactorily adequate probability of obtaining an upper confidence limit under H_0_ and a lower confidence limit under H_1_, both bounded by fixed cut-offs, thus making it possible to declare a definitely positive or definitely negative result. The authors deserve being complimented on their paper, but we believe some points should be considered further.

According to Jia and Lynn [1], “sample size needs to be increased 4-fold when comparing normally distributed means” and four to five times “when evaluating the log-hazard ratio for time-to-event data”; this increase raises substantial doubts concerning the real feasibility of phase II/III clinical trials and, consequently, the practical usefulness of the method. Indeed, the sample sizes should be as small as possible not only because “in practice, cost constraints force clinical trials to aim for the smallest possible sample size” but (and more importantly) because an ethical imperative exists to ensure that the number of patients exposed to a treatment that proves to be statistically inferior at the end of a controlled clinical trial is minimized.

Most statistical methods implemented in controlled clinical trials (CCTs) have the aim of reducing the number of enrolled patients. This aim not only meets the a priori imperative of exposing the minimum number of patients to the burdens of a trial [2] but also fulfills the a posteriori imperative that as few patients as possible are administered the treatment that proves to be inferior. These ethical requirements also underlie the introduction of group sequential designs insofar as a CCT can only be carried out if the investigators are equipoised.

In brief, to administer patients a potentially less effective treatment only for the purpose of having a highly precise confidence interval (CI) and/or arriving at a “definite conclusion” concerning efficacy is not possible.

Jia and Lynn’s proposal of drawing “attention back to the importance of gauging effect sizes using confidence intervals” may be considered in the case of a randomized phase IV trial aimed at assessing a drug’s effectiveness on a continuous variable or hazard ratio as the primary outcome. However, phase IV trials are not usually randomized or based on a precise estimate of the prevalence of a rare serious adverse event.

The joint aim of obtaining power and precision with an acceptable increase in a power-based sample size (thus making a CCT ethically and economically feasible) can be achieved using our proposal [3], which is based on the first research priority of demonstrating a clinically relevant difference between treatments. This approach considers the precision of the effect estimate by calculating the standardized expected half-width (EH) of the CI obtained by the power-based sample size and the probability of obtaining standardized half-widths of sample CIs that are less than the EH, conditional on the coverage (P(EH|C)). In addition, the approach makes it possible to take into account a very broad scenario of precise estimation by calculating various values of standardized half-widths (Hj) and the probability of obtaining sample standardized half-widths that are less than Hj, conditional on the coverage (P(Hj|C)). Subsequently, by iteratively increasing the starting power-based sample size, the achievement of an adequate value (at least 0.80) of the joint probability function combining the power of the statistical test and the power of the confidence interval (P(EH|C)) is possible by increasing the sample size by about 20 %.

Furthermore, according to the International Conference for Harmonization (ICH) Guidance E9 [4], “The number of subjects in a clinical trial should always be large enough to provide a reliable answer to the questions addressed,” and therefore, underpowered CCTs should always be avoided.

Finally, we think the best approach to sample size calculation should simultaneously fulfill the two research requirements of having an adequate probability of demonstrating a difference (power of the statistical test) and being capable of estimating it as precisely as possible (power of the CI). Therefore, we believe it is sensible to start from the EH derived from the power-based sample sizes, which should be considered the precision threshold given the foreseen difference under H_1_.

## Abbreviations

CCTs: controlled clinical trials
CI: confidence interval
EH: standardized expected half-width
Hj: standardized half-widths
H_0_: null hypothesis
H_1_: alternative hypothesis
ICH: International Conference for Harmonization
P(EH|C): probability of obtaining standardized half-widths of sample CIs that are less than the EH, conditional on the coverage
P(Hj|C: probability of obtaining standardized half-widths that are less than Hj, conditional on the coverage
